# Locus coeruleus inhibition of vibrissal responses in the trigeminal subnucleus caudalis are reduced in a diabetic mouse model

**DOI:** 10.3389/fncel.2023.1208121

**Published:** 2023-07-05

**Authors:** Alberto Mesa-Lombardo, Nuria García-Magro, Angel Nuñez, Yasmina B. Martin

**Affiliations:** ^1^Department of Anatomy, Histology and Neurosciences, Universidad Autónoma de Madrid, Madrid, Spain; ^2^Facultad de Ciencias de la Salud, Universidad Francisco de Vitoria, Pozuelo de Alarcón, Madrid, Spain; ^3^Facultad de Medicina, Universidad Francisco de Vitoria, Pozuelo de Alarcón, Madrid, Spain

**Keywords:** neuropathic pain, noradrenergic transmission, diabetes, α2-noradrenergic receptor, clonidine, yohimbine, hyperglycemia

## Abstract

Diabetic neuropathy is the loss of sensory function beginning distally in the lower extremities, which is also characterized by pain and substantial morbidity. Furthermore, the locus coeruleus (LC) nucleus has been proposed to play an important role in descending pain control through the activation of α2-noradrenergic (NA) receptors in the spinal dorsal horn. We studied, on control and diabetic mice, the effect of electrical stimulation of the LC nucleus on the tactile responses in the caudalis division of the spinal trigeminal nucleus (Sp5C), which is involved in the relay of orofacial nociceptive information. Diabetes was induced in young adult C57BL/6J mice with one intraperitoneal injection of streptozotocin (50 mg/kg) daily for 5 days. The diabetic animals showed pain in the orofacial area because they had a decrease in the withdrawal threshold to the mechanical stimulation in the vibrissal pad. LC electrical stimulation induced the inhibition of vibrissal responses in the Sp5C neurons when applied at 50 and 100 ms before vibrissal stimulation in the control mice; however, the inhibition was reduced in the diabetic mice. These effects may be due to a reduction in the tyrosine hydroxylase positive (TH+) fibers in the Sp5C, as was observed in diabetic mice. LC-evoked inhibition was decreased by an intraperitoneal injection of the antagonist of the α2-NA receptors, yohimbine, indicating that it was due to the activation of α2-NA receptors. The decrease in the LC-evoked inhibition in the diabetic mice was partially recovered when clonidine, a non-selective α2-agonist, was injected intraperitoneally. These findings suggest that in diabetes, there is a reduction in the NA inputs from the LC in the Sp5C that may favor the development of chronic pain.

## 1. Introduction

Diabetic neuropathy is the loss of sensory function beginning distally in the lower extremities, which is also characterized by pain and substantial morbidity. It is a neurodegenerative disorder of the peripheral nervous system that preferentially targets sensory axons, autonomic axons, and later, to a lesser extent, motor axons (Vincent et al., 2011; Feldman et al., 2019). Neuropathic pain is one of the most prevalent complications of diabetes. Approximately 30–50% of patients with diabetic neuropathy develop neuropathic pain (Abbott et al., 2011; Feldman et al., 2019; Rastogi and Jude, 2021). The locus coeruleus (LC) nucleus is proposed to play an important role in descending pain control (Jones, 1991; Millan, 2002; Hickey et al., 2014; Schwarz and Luo, 2015; Benarroch, 2018; Hayashida and Obata, 2019; Ross and Van Bockstaele, 2020). However, its participation in pain control in diabetic neuropathy is not well known in the trigeminal complex.

The LC neurons are the major source of noradrenaline with profuse projections across the central nervous system, including the brain stem nuclei (Sasa and Takaori, 1973; Loughlin et al., 1986; Aston-Jones, 2005; Shiraishi et al., 2021). The LC neurons are implicated in a variety of brain functions, including sleep–wake control, sensory perception, attention, and learning. The descending noradrenergic (NA) inhibitory system is one of the main pathways in endogenous pain modulation (Yaksh, 1989; Millan, 2002; Pertovaara, 2006; Donertas-Ayaz and Caudle, 2023). Patients with trigeminal neuropathy had altered LC functional connectivity with increased connectivity between the rostral ventromedial medulla and decreased connectivity between the ventrolateral periaqueductal gray matter (Mills et al., 2018). Stimulation of the LC produces profound antinociception in the spinal dorsal horn *via* stimulation of the α2-NA receptors, which are coupled with the inhibitory G protein (Jones, 1991; West et al., 1993; Sonohata et al., 2004). In chronic neuropathic pain models, there is an increase in the NA innervation of the spinal dorsal horn (Ma and Eisenach, 2003; Hughes et al., 2013) and an increase in α2-adrenoceptor sensitivity (Ma and Eisenach, 2003; Hayashida and Eisenach, 2008). Accordingly, clonidine, a non-selective α2-NA receptor agonist, is effective in alleviating neuropathic pain in humans (Eisenach et al., 1995) or in experimental animals (Puke and Wiesenfeld-Hallin, 1993).

The caudalis division of the spinal trigeminal nucleus (Sp5C) is generally considered to be involved in the relay of orofacial nociceptive information (Hu, 1990; Bae et al., 2000; Bereiter et al., 2000; Martin et al., 2010). Anatomical evidence indicates that the LC sends NA projections to the trigeminal brainstem sensory nuclear complex (Levitt and Moore, 1979). Electrophysiological studies have also shown that the spontaneous neuronal activity and responses of Sp5C neurons to the somatic and nociceptive inputs are inhibited by stimulation of the LC (Sasa and Takaori, 1973; Tsuruoka et al., 2003). Thus, it is reasonable to believe that diabetes may affect the descending NA inhibitory system on the Sp5C neurons, contributing to the development of chronic pain. In this context, the main goal of the present study was to examine whether diabetes induced by streptozotocin (STZ) injections altered the response properties of the Sp5C neurons to vibrissal stimulation and whether the LC inhibitory effects were affected in STZ-induced diabetes in anesthetized mice.

## 2. Materials and methods

### 2.1. Animals

The experiments were performed using young adult (2–3 months old) male C57BL/6J mice (*n* = 58; weight ranged between 22 and 28g, Harlan Laboratories, Spain). The mice were placed in two groups, STZ-induced diabetic mice (*n* = 25) and control mice (*n* = 33) that only received vehicles. All the mice were housed under a 12:12-h dark/light cycle at 22 ± 2°C with food and water *ad libitum*. All the animal procedures followed the European guidelines (2010/63, European Council Directives) and were approved by the local Ethical Committee (Autonomous University of Madrid and Government of the Community of Madrid; PROEX: 181.6/21). Efforts were made to minimize animal suffering as well as to reduce the number of mice used. A double-blinding strategy was performed in all steps of the experiment. A technician oversaw measuring glucose and took care of the animals while the researcher carried out the behavioral, anatomical studies, or electrophysiological recordings without knowing which group the mouse belonged to.

### 2.2. STZ-dependent diabetes

STZ is an antibiotic that produces pancreatic islet β-cell destruction and is widely used experimentally to produce a model of type 1 diabetes mellitus (Furman, 2021). This animal model is employed for assessing the pathological consequences of diabetes and for screening potential therapies for the treatment of this condition. We used the protocol published by Perez-Taboada et al. (2020) to induce diabetes in mice (Perez-Taboada et al., 2020). STZ (50 mg/kg, intraperitoneally, i.p.; Sigma-Aldrich, St. Louis, MO) was administered for 5 consecutive days. The mice were considered diabetic when their glucose levels, as monitored using a glucometer (Glucoleader-Yasee GLM-76, Nessler, Spain), using tail blood after a 4-h fast, were >300 mg/dl. The control mice were injected with the vehicle (10 mM sodium citrate, 0.9% NaCl; pH 4.5, i.p.). Glucose measurements were performed prior to the STZ injection, throughout the 3 weeks of diabetes development, before the experimental recordings, or before each of the behavioral tests.

### 2.3. Behavioral test

An assessment of the response to mechanical allodynia was performed using a series of six calibrated nylon von Frey monofilaments (North Coast Medical, Inc., Morgan Hill, CA, USA), with a bending force ranging from 0.008 to 0.4 g. Before 3 days of starting the experiments and before every stimulation session, the mice were acclimatized to the testing room for ~1 h daily. The stimuli were applied bilaterally in different areas of the vibrissal pad, according to the procedure described by Krzyzanowska et al. (Krzyzanowska et al., 2011). The experiments started 3 consecutive days before either the STZ or vehicle injection to establish the baseline response and on post-injection days 14 and 21 (see **Figure 5A**). The von Frey filaments were applied in ascending order five times on five different points of each vibrissal pad. Each probe was applied until it just bent. The time interval between consecutive filament administrations was at least 5 s (Vos et al., 1994). The response threshold was considered as the lowest force of the filaments that produced a brisk head withdrawal in more than 50% of trials (3 out of 5).

### 2.4. Recordings and tactile stimulation

The mice were anesthetized with isoflurane (2% induction; 1–1.5% maintenance doses) and placed in a David Kopf stereotaxic apparatus (Tujunga, CA, USA). Body temperature was set at 37°C through a water-heated pad (Gaymar T/Pump, Orchard Park, NY, USA). The skin over the midline of the scalp was sectioned and retracted. A small craniotomy was drilled over the LC nucleus according to the atlas of Paxinos and Franklin (coordinates from Bregma: A: −5.4 mm, L: 0.9 mm lateral, H: 3.5 mm; (Paxinos and Franklin, 2003).

Tungsten microelectrodes (2 MW) were used to obtain single-unit recordings in the Sp5C (A: −7.6 mm, L: 2 mm from bregma; H: 0.5–1.5 mm from the surface of the nucleus). The recording electrode was introduced at a 60° angle to the surface of the nucleus after opening the cisterna magna. The position of the electrodes was visually controlled under a dissecting microscope. The unit recordings were filtered between 0.3 and 3 kHz and amplified using a DAM50 preamplifier (World Precision Instruments, Friedberg, Germany). The signals were sampled at 10 kHz through an analog-to-digital converter (Power 1401 data acquisition unit, Cambridge Electronic Design, Cambridge, UK) and fed into a PC for offline analysis with Spike 2 software (Cambridge Electronic Design).

Vibrissal deflections were evoked by brief air pulses using a pneumatic pressure pump (Picospritzer, Hollis, NH, USA; 1–2 kg/cm^2^, 20 ms duration), delivered through a 1 mm inner diameter polyethylene tube. The experimental protocol consisted of air pulses delivered to the vibrissal at 0.5 Hz for 1 min (30 stimuli) for basal recording before pair-pulse stimulation in the LC nucleus and vibrissal at 50–300 ms delays for 1 min (30 pair of stimuli). After the pair-pulse protocol, the vibrissal was stimulated for 1 min at 0.5 Hz (30 stimuli) to test if the vibrissal response recovered from the effect of LC stimulation.

### 2.5. Drugs

The antagonist of α2-NA receptors yohimbine (2 mg/Kg i.p.; Sigma, St Louis, MO, USA) or the non-selective α2 agonist clonidine (2 mg/Kg i.p.; Sigma, St Louis, MO, USA) was i.p. administrated 30 min before the recording session. We have selected these doses according to previous findings (Tank et al., 2004).

### 2.6. Immunohistochemistry

The mice were deeply anesthetized (Dolethal, 50 mg/kg i.p. Vétoquinol; Madrid, Spain) and perfused transcardially with saline 0.9% followed by 4% paraformaldehyde in 0.1 M phosphate buffer (PB; pH 7.4). The brainstem was extracted and the trigeminal ganglion (TG) of both sides was excised by sectioning the trigeminal root and the three trigeminal branches. All tissues were postfixed in the same fixative overnight and cryoprotected for 2 days in 30% sucrose in PB. Coronal sections of the brainstem 40-μm-thick were cut serially using a sliding microtome (Leica SM2400, Leica Biosystems, Nussloch) and collected in PB 0.1 M. All sections were processed free-floating. The sections were incubated in a blocking solution (PB 0.1 M, 1% Triton X-100 and donkey normal serum 10%) for 2 h at room temperature, followed by incubation at 4°C with primary monoclonal antibody rabbit anti-TH (1:2000; ab137869, Abcam) in a blocking solution for 24 h. After washing three times in PB 0.1 M, sections were incubated with a polyclonal secondary antibody donkey anti-rabbit AlexaFluor 488 (1:200, Molecular Probes, USA) for 2 h in the dark at room temperature. The TG was frozen and serially cut at 15 μm in a Leica CM1950 cryostat (Leica Biosystems, Nussloch, GmbH). TH fluorescent labeling of every third section was performed following the same protocol as the brainstem on slides using a wet chamber.

Finally, after two washes in PB, 1:3,000 dilution in PB of Hoechst (Thermo Fisher Scientific; Waltham, MA, USA) was added for 5 min to label the nuclei. The sections were then mounted on glass slides and coverslips with Prolong^TM^ (Thermo Fisher Scientific, Waltham, MA, USA).

### 2.7. Microscopy and image analysis

Confocal microscopy images of the LC, TG, and Sp5C on both sides were obtained using a TCS SP5 Spectral Leica confocal microscope (Leica Mycrosystems AG; Wetzlar, Germany) using a 40 X (Sp5C) or 20 X (LC and TG) oil immersion objectives, respectively. Image stacks were acquired at 1024 x 1024 pixels using Leica LAS AF software.

The analysis was carried out using the ImageJ image analysis software for Windows (Microsoft; Albuquerque, NM, USA). Images obtained by confocal microscopy were collapsed to create TIFF files with the projections of maximum intensity using the same final tissue thickness analyzed for all series (10 μm). To ensure comparable immunostaining, the sections were processed together under the same conditions. A threshold was set to remove the background. The region of interest was delineated using an ROI that included the LC and, in the case of Sp5C, two broad regions of interest including, respectively, lamina II and laminae III-IV and densitometric analysis of TH immunoreactivity, and performed on each image, obtaining optical density measurements using the ImageJ 'Set Measurement' routine. Similarly, optical density measurements were obtained from the TG, using a specific ROI applied to all images taken that cover each of the sections in their entirety. These gray values were used to compose the histograms and to perform the statistical analysis.

### 2.8. Statistical analysis

The peristimulus time histograms (PSTHs) were used to calculate spike responses in a 50 ms post-stimulus time window following each stimulus (1 ms bin-width). The mean response during the basal recording was considered to be 100%, and the effect of LC stimulation during the pair-pulse stimulation protocol was calculated.

Statistical analysis was performed using Graph Pad Prism 9 software (San Diego, CA, USA). Any differences between the variables were compared using a two-way parametric (Student's t-test or paired t-test) after normality testing (Kolmogorov–Smirnov normality test). The sample size for each experiment was chosen based on previous experience. Data were expressed as the mean ± standard error of the mean (SEM) with n indicating the number of mice per group for a given experiment or the number of neurons analyzed. The results were considered significant at a p-value of < 0.05 (^*^p < 0.05, ^**^p < 0.01, ^***^p < 0.001).

## 3. Results

### 3.1. Reduction of NA signaling in diabetic mice

**Figure 1:**
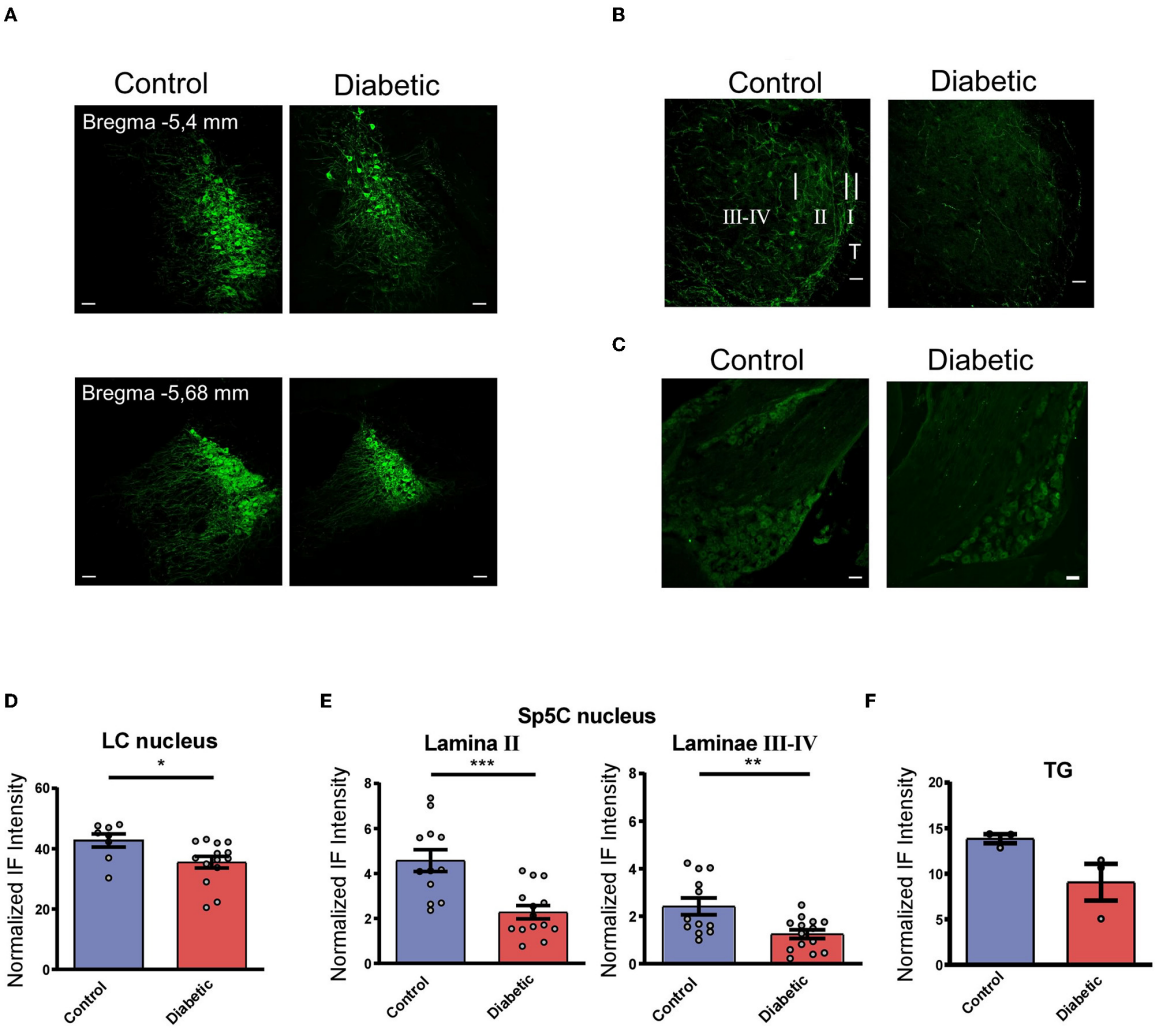
Diabetic mice showed a reduction in their TH+ cells in LC and TH+ terminals in Sp5C. **(A)** Representative photomicrographs of TH+ labeled cells in the LC of the control (left) and the diabetic mice (right). Two different anteroposterior planes are shown [−5.4 mm (upper) and −5.68 mm (lower) from bregma photographs; 20 X]. Note the reduction in the TH+ labeled cells in diabetic mice. **(B)** Representative photomicrographs of TH+ labeled fibers in the Sp5C of the control (left) and diabetic mice (right) at −7.6 mm from bregma (40X). A clear reduction in TH+ fibers is observed in lamina II and laminae III–IV in diabetic mice. In the control section (left) short, white bars indicate the approximate boundaries between the trigeminal tract (T) and lamina I (I), lamina II (II), and laminae III-IV (III–IV). These sections were among those used for densitometric measures. **(C)** Representative photomicrographs of TH+ labeled cells in the TG of the control (left) and the diabetic mice (right). These sections were among those used for densitometric measures. **(D–F)** Density measures of TH+ immunoreactivity in LC **(D)**, lamina II, and laminae III-IV in the Sp5C **(E)** and TG **(F)**. The density measures showed statistically significant differences between the control and the diabetic mice (*n* = 4 and 7, respectively, using hemispheres as sampling units) in all cases (*p* = 0.0269 in LC; *p* = 0.0003 in SP5C lamina II; *p* = 0.0053 in Sp5C laminae III-IV). Scale bar 25 μm **(A)** and 50 μm **(B, C)**. In this and in the following figures *p < 0.05; **p < 0.01; ***p < 0.001.

The immunohistochemical studies were focused on the LC and Sp5C nuclei in order to study the differences in the LC projections to Sp5C between the control and the diabetic mice. The immunohistochemistry of the tyrosine hydroxylase (TH), which is the enzyme responsible for the synthesis of NA, showed abundant staining in the LC cells of the control and the diabetic mice measured on both sides (Figure 1A). The control mice showed a higher expression of TH positive (TH+) cells at all rostro-caudal levels of the LC nucleus than the diabetic mice. The optical density of the normalized fluorescence intensity indicated a statistically significant reduction in the TH+ neurons in the diabetic mice with respect to the controls (*p* = 0.0269; *n* = 7 and 4 mice, respectively; unpaired *t*-test; Figure 1D).

In addition, TH+ fibers were present in all laminae of the Sp5C nucleus of the control and diabetic mice (Figure 1B). A few TH+ neuronal bodies could be observed; however, their number was similar in control or diabetic animals. TH+ fibers were strongly expressed as a homogeneous band in lamina II, and more moderately in laminae III-IV in both control and diabetic animals. The control mice showed a higher expression of TH+ fibers while a clear reduction in TH+ fibers was observed in the diabetic mice in all laminae. A densitometric analysis of both sides showed a clear reduction of immunofluorescence intensity, reached statistical significance, in TH+ expression in the diabetic compared with the control mice (*n* = 7 and 4 mice, respectively) in lamina II (*p* = 0.0003) and in laminae III-IV (*p* = 0.0053; unpaired *t*-test; Figure 1E).

TG immunolabeled TH+ cells are distributed similarly in both control and diabetic animals (Figure 1C); however, densitometric measurements show a decrease in immunolabeling intensity in diabetic mice although it does not reach statistical significance (*p* = 0.0836; Figure 1F). In summary, this indicated that there was a decrease in NA inputs in the Sp5C of the diabetic mice that may implicate a reduction in the LC control of the trigeminal responses.

### 3.2. LC stimulation inhibited vibrissal responses of Sp5C neurons in the control but not in the diabetic mice

The electrical stimulation of LC induced an orthodromic response in Sp5C neurons of 1.4 ± 0.23 spikes/stimulus (*n* = 31 neurons), with a mean latency of 6.4 ± 0.2 ms. The Sp5C neurons from the diabetic mice also responded to LC electrical stimulation with an orthodromic response of 1.3 ± 0.25 spikes/stimulus (*n* = 25 neurons) and with a mean latency of 6.8 ± 0.4 ms (*n* = 25 neurons). No statistical differences were found with respect to control animals (*p* > 0.05). Vibrissal stimulation evoked similar responses in both the control and the diabetic mice (2.5 ± 0.14 spikes/stimulus; *n* = 37 neurons or 2.5 ± 0.1 spikes/stimulus; *n* = 25 neurons, respectively; *p* > 0.05; unpaired test in all these cases).

**Figure 2:**
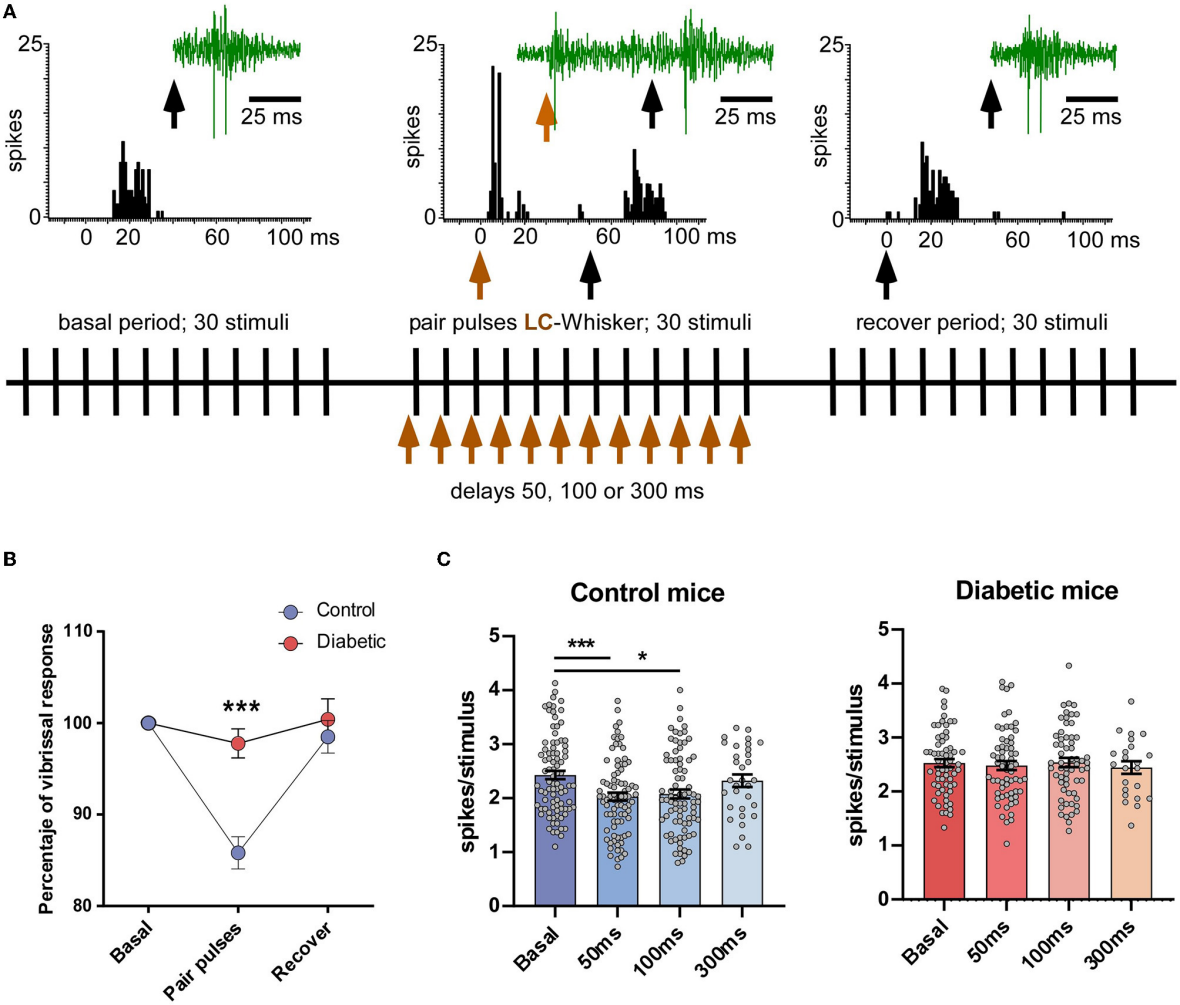
LC electrical stimulation induced an inhibition of the vibrissal responses in the control mice but not in the diabetic mice. **(A)** A scheme of the stimulation protocol is shown. The upper insets show representative PSTHs of the vibrissal responses (30 stimuli) in a control case and an example of the unit recordings (green traces). The black vertical arrows indicate vibrissal stimulation; the brown vertical arrow indicates LC stimulation. The vibrissal response was reduced when LC stimulation occurred 50 ms before vibrissal stimulation. **(B)** Plot of the percentage of LC-evoked inhibition in the control and the diabetic animals when LC stimulation preceded 50 ms vibrissal stimulation. **(C)** Plots of the mean vibrissal response in a basal period and when the LC and vibrissal stimulation were paired at 50, 100, and 300 ms. The response was reduced in the control mice (left plot) at a 50 and 100 ms delay. By contrast, LC stimulation did not affect the vibrissal responses at any delay in the STC-induced diabetic mice (right plot). In this and in the following figures *p < 0.05; ***p < 0.001.

To test the effect of LC stimulation on the vibrissal responses, a pair stimulation protocol was used (Figure 2A). LC stimulation inhibited vibrissal responses when LC stimulation preceded vibrissal stimulation for 50 ms in the control mice. When LC stimulation preceded 50 ms vibrissal stimulation, it evoked a reduction in the vibrissal responses from 2.4 ± 0.08 spikes/stimulus to 2.0 ± 0.07 spikes/stimulus (*p* < 0.001; *n* = 84 neurons; 17%; paired test). However, in the diabetic mice, LC stimulation did not reduce the vibrissal responses at a 50 ms delay (from 2.5 ± 0.07 spikes/stimulus to 2.4 ± 0.08 spikes/stimulus; *p* > 0.05; *n* = 63 neurons; 96%; Figure 2B). The vibrissal response was also reduced when LC stimulation preceded 100 ms vibrissal stimulation (2.1 ± 0.09 spikes/stimulus; *p* = 0.0116; *n* = 82 neurons; paired test; Figure 2C, left plot). However, the vibrissal responses were not affected when the delay was 300 ms (2.3 ± 0.11 spikes/stimulus; *p* > 0.05; *n* = 32 neurons; 4.0%; paired test; Figure 2C, left plot). In the diabetic mice, LC stimulation did not reduce the vibrissal responses at any delay (2.5 ± 0.08 spikes/stimulus; *p* > 0.05; *n* = 62 neurons at 100 ms delay and 2.4 ± 0.11 spikes/stimulus; *p* > 0.05; *n* = 23 neurons, at 300 ms delay; Figure 2C, right panel).

### 3.3. Mechanisms of LC-evoked inhibition in the Sp5C neurons

It has been established that LC sends NA projections to the trigeminal complex (Sasa and Takaori, 1973; Levitt and Moore, 1979). Thus, we studied if the antagonist of the α2-NA receptors, yohimbine (2 mg/Kg i.p.), or the non-selective α2-NA agonist clonidine (2 mg/Kg i.p.) affected the LC or vibrissal responses as well as the LC-evoked inhibition of the Sp5C neurons.

**Figure 3:**
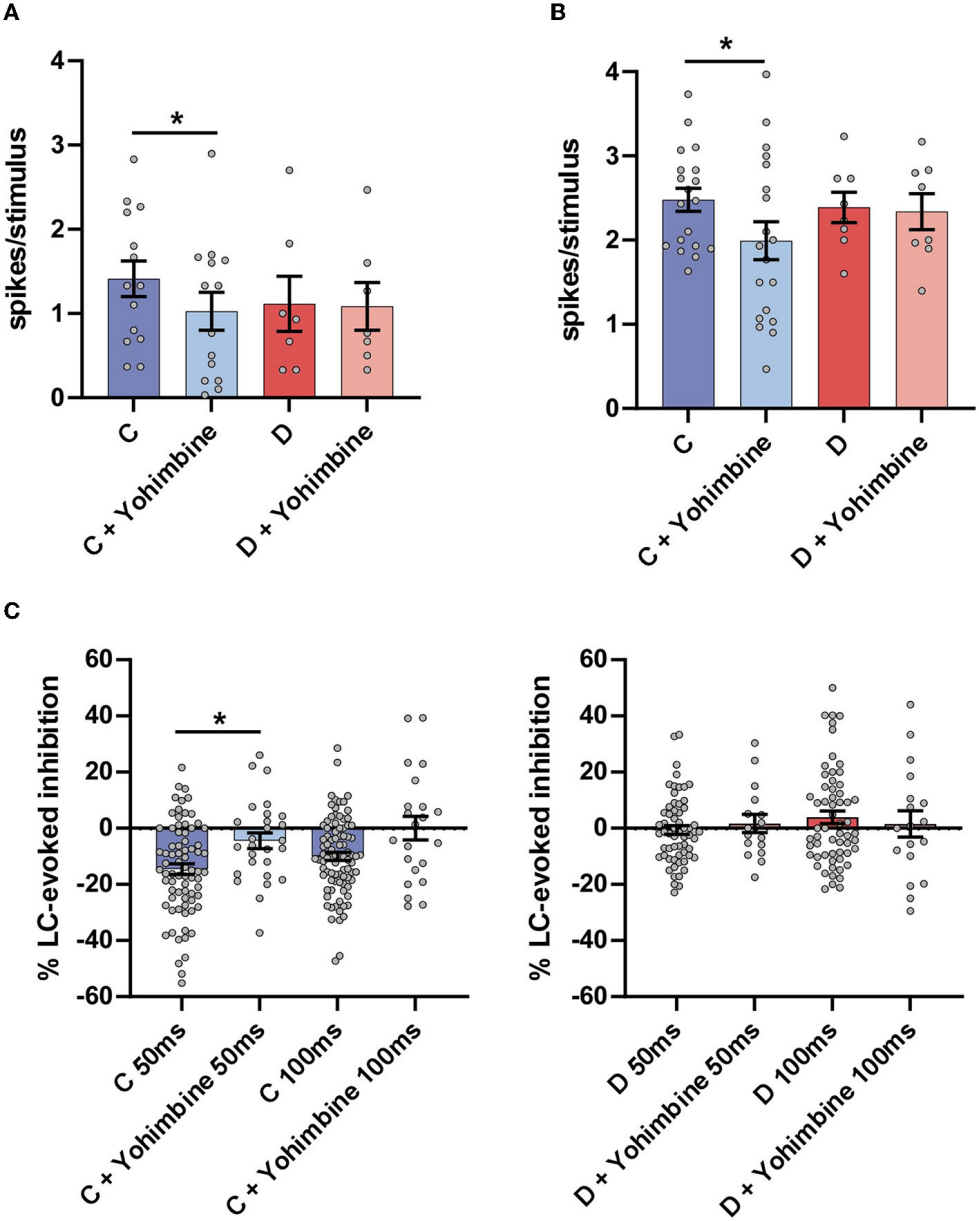
Intraperitoneal injection of the antagonist of the α2-NA receptors yohimbine reduced the LC and vibrissal responses only in the control mice as well as the LC-evoked inhibition. **(A)** Plot of the mean LC response in the control (C; blue bars) and in the diabetic (D; red bars) mice. The LC response in the basal condition (before drug injection; strong blue color) was reduced after yohimbine injection (2 mg/kg i.p. light blue bar). However, the LC responses were not affected by yohimbine in the diabetic mice (strong and light red bars). **(B)** Plot of the mean vibrissal response in the control (blue bars) and in the diabetic (red bars) mice. In the control mice, the vibrissal response was reduced after the yohimbine injection but not in the diabetic mice. **(C)** Plots of the LC-evoked inhibition at a 50 and a 100 ms delay. Yohimbine injection reduced the LC-evoked inhibition in the control mice but did not affect the inhibition in the diabetic mice. ^*^p < 0.05.

Intraperitoneal injection of yohimbine significantly reduced the orthodromic responses evoked by LC stimulation in the control mice (from 1.4 ± 0.21 spikes/stimulus to 1.0 ± 0.22 spikes/stimulus; n = 14 neurons; p = 0.0159; paired *t*-test) but not in the diabetic mice (from 1.1 ± 0.22 spikes/stimulus to 1.1 ± 0.28 spikes/stimulus; n = 7 neurons; p > 0.05; paired *t*-test; Figure 3A). Yohimbine also reduced the orthodromic responses evoked by vibrissal stimulation in the control mice from 2.5 ± 0.13 spikes/stimulus to 1.9 ± 0.22 spikes/stimulus (*n* = 19 neurons; *p* = 0.0163; paired *t*-test) but not in the diabetic mice (from 2.4 ± 0.18 spikes/stimulus to 2.3 ± 0.21 spikes/stimulus; *n* = 8 neurons; p > 0.05; paired *t*-test; Figure 3B). In addition, the LC-evoked inhibition of vibrissal responses at a 50 ms delay in the control animals was reduced from 14.5 ± 1.9% of inhibition to 4.5 ± 2.8% (*n* = 27 neurons; *p* = 0.023), 30 min after yohimbine application (Figure 3C, left plot). The same occurred when the delay between LC and vibrissal stimulation was 100 ms. The LC-evoked inhibition was reduced from 9.9 ± 1.4% inhibition to 1.1 ± 5.6% facilitation although the differences were not statistically significant (*n* = 24 neurons; *p* > 0.05; paired *t*-test), indicating that the LC-evoked inhibition in Sp5C was mainly due to the activation of the α2-NA receptors. In the diabetic mice, the LC-evoked inhibition of the vibrissal response was very low in basal conditions at 50 ms delay (−1.6 ± 1.7%; *n* = 16 neurons) and changed to slight facilitation after the yohimbine i.p. injection (+1.7 ± 3.2%; Figure 3C, right plot). At 100 ms delay, the slight facilitation observed in the basal conditions changed to slight inhibition (from +3.9 ± 2.2% to −0.6 ± 4.9%; *n* = 19 neurons); the differences in the diabetic mice were not statistically significant (*p* > 0.05).

**Figure 4:**
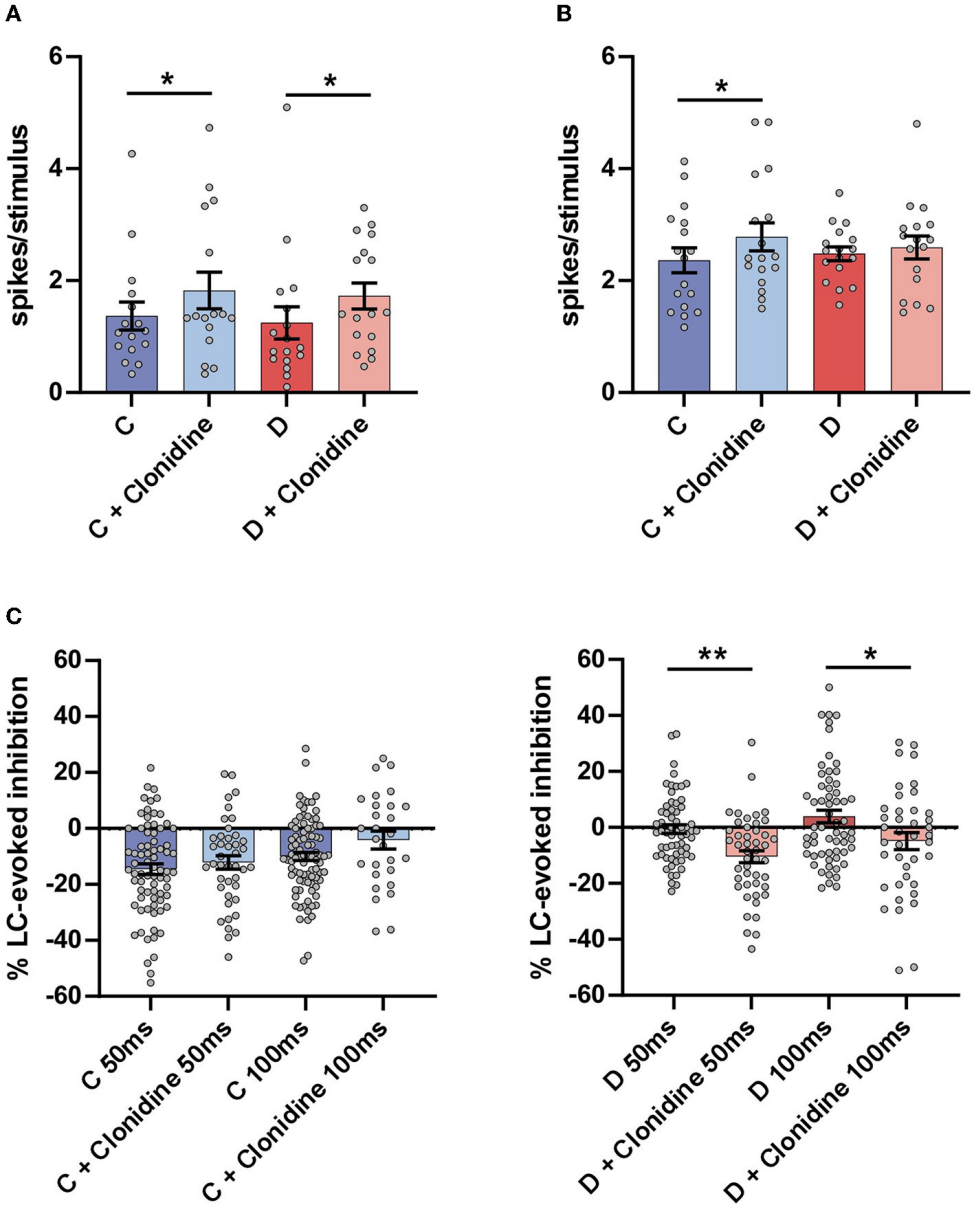
Effect of the non-selective α2 agonist clonidine on Sp5C responses. **(A)** Plot of the mean LC responses in the control (C; blue bars) and in the diabetic (D; red bars) mice. The LC response (strong blue bar before drug injection) was increased after clonidine injection in the control mice (2 mg/Kg i.p.; light blue bar). The increment of LC responses in the diabetic mice did not reach statistical significance (strong and light green bars). **(B)** Plot of the mean vibrissal response in the control (blue bars) and in the diabetic (green) mice. In the control mice, the vibrissal response was increased after clonidine injection but not in the diabetic mice. **(C)** Plots of the LC-evoked inhibition at a 50 and a 100 ms delay. The clonidine injection reduced the LC-evoked inhibition slightly in the control mice, but these differences were not statistically significant. However, the LC-evoked inhibition was increased significantly in diabetic mice. ^*^p < 0.05, ^**^p < 0.001.

The i.p. injection of the α2-NA agonist clonidine increased the orthodromic responses evoked by LC stimulation in the control mice from 1.4 ± 0.25 spikes/stimulus to 1.8 ± 0.33 spikes/stimulus (*n* = 16 neurons; *p* = 0.0231; paired *t*-test) and in the diabetic mice (from 1.2 ± 0.29 spikes/stimulus to 1.7 ± 0.23 spikes/stimulus; n = 17 neurons; p = 0.0326; Figure 4A). Clonidine also increased the orthodromic responses evoked by vibrissal stimulation in the control mice, from 2.3 ± 0.22 spikes/stimulus to 2.8 ± 0.25 spikes/stimulus (*n* = 17 neurons; *p* = 0.0116; paired t-test) but not in the diabetic mice from 2.5 ± 0.12 spikes/stimulus to 2.6 ± 0.21 spikes/stimulus (*n* = 17 neurons; *p* > 0.05; Figure 4B).

The i.p. injection of clonidine did not affect LC-evoked inhibition in the control mice at a 50 ms delay (from 14.1 ± 1.7% to 12.2 ± 2.4%; *n* = 41 neurons; p >0.05) or at a 100 ms delay (from 9.8.1 ± 1.4% to 2.4 ± 3.6%; *n* = 29 neurons; *p* > 0.05) although the response of the LC stimulation increased (see above; Figure 4C, left plot). However, in the diabetic mice in which the LC-evoked inhibition was very low at a 50 ms delay in the basal condition, it increased after the injection of clonidine (from 1.7 ± 1.5% to 10.5 ± 2.2%; *n* = 46 neurons; *p* = 0.0015; paired *t*-test). At a 100 ms delay, LC facilitated the vibrissal response in the basal conditions, and this effect changed to slight inhibition after the clonidine injection in the diabetic mice (from +4.2 ± 2.5% to −4.9 ± 3.0%; *n* = 46 neurons; *p* = 0.0189; paired *t*-test; Figure 4C, right plot), suggesting that the α2-NA receptors remained active in the diabetic mice and recovered the inhibitory action of LC. Taken together, these results indicate that LC inhibited vibrissal responses in the Sp5C by the activation of α2-NA receptors. In diabetic mice, LC-evoked inhibition was reduced, but the α2-NA receptors remained active because clonidine could increase this inhibitory effect. Consequently, the reduction in the LC-evoked inhibition in the diabetic mice induced changes in the responsiveness to mechanical stimuli and, therefore, in the pain sensation.

### 3.4. Pain test in diabetic mice

**Figure 5:**
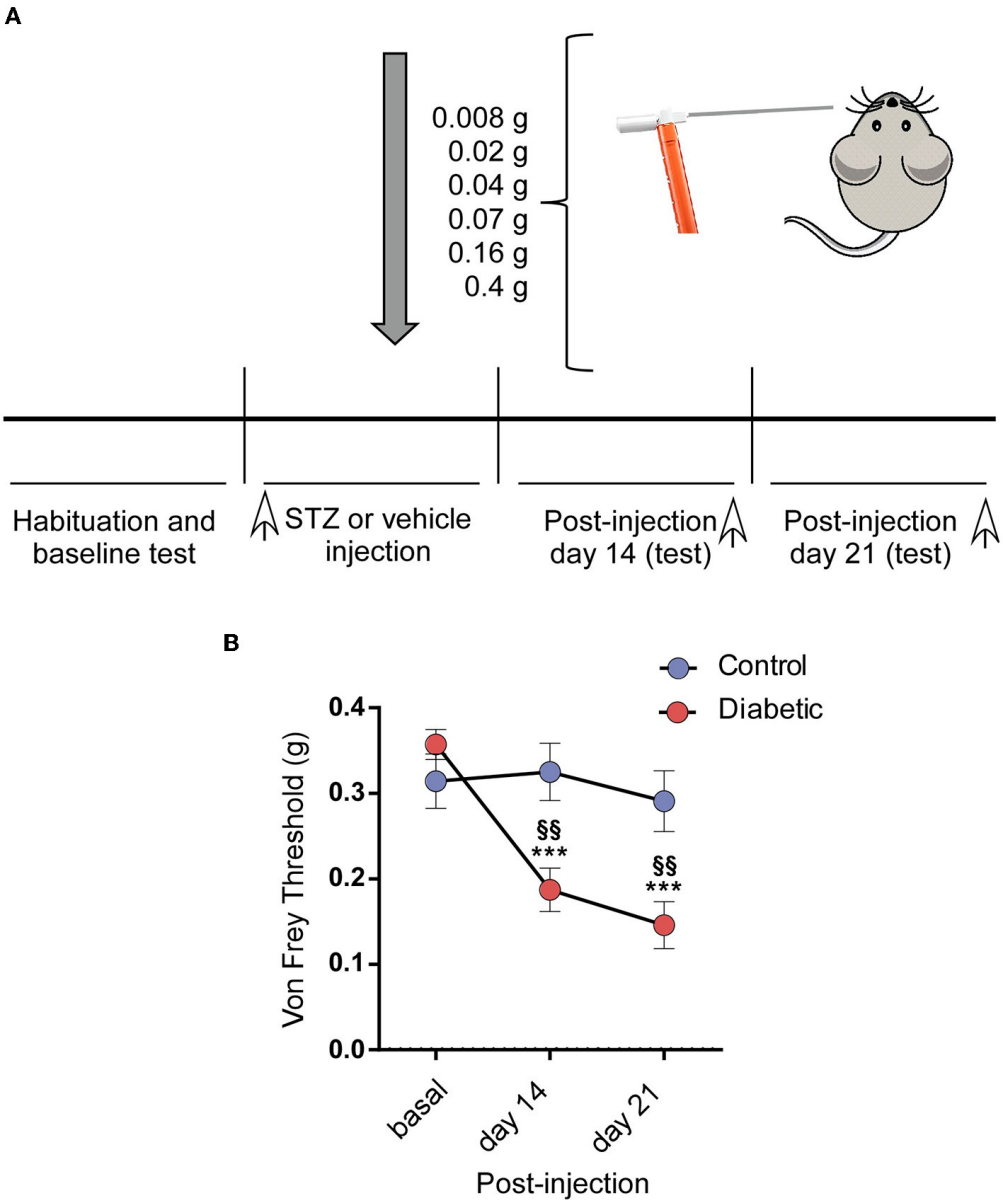
Diabetic mice showed pain in the orofacial area. **(A)** A scheme of the behavioral test is shown. **(B)** The diabetic mice showed a decrease in the withdrawal threshold to mechanical stimulation in the vibrissal pad the 2nd and 3rd week after the STZ injection by comparison with the basal values obtained before the STZ injection. Asterisks indicate the differences with the basal values; §§ differences with respect to the values obtained in the control mice. ***p < 0.001; §§*p* < 0.01.

The responsiveness to mechanical stimuli was assessed in the second and third week after STZ injection and compared with control values obtained in the baseline before STZ injection (Figure 5A). The diabetic mice showed a marked decrease in the Von Frey withdrawal threshold to mechanical stimulation in the vibrissal pad. We did not observe any change in the threshold with time in the control mice (0.31 ± 0.032 g; 0.32 ± 0.033 g; 0.29 ± 0.035 g; *n* = 14 animals; Figure 5B). However, the withdrawal threshold decreased at 2 or 3 weeks compared with that observed in their basal conditions (0.35 ± 0.018 g; 0.19 ± 0.025 g; 0.15 ± 0.027 g; *n* = 28 animals; *p* < 0.0001 in both cases, with respect to their basal values; paired *t*-test; Figure 5B). The mechanical response threshold in the diabetic mice was also different at 2 or 3 weeks in comparison with that observed in the controls (*p* = 0.0027 or *p* = 0.0033, respectively; unpaired *t*-test).

## 4. Discussion

The involvement of LC in the control of pain and tactile detection was shown decades ago (Cedarbaum and Aghajanian, 1976; Jones, 1991; Pertovaara, 2006; Mcburney-Lin et al., 2019). The present results have demonstrated that the inhibition elicited by LC stimulation in the Sp5C nucleus was reduced in diabetic mice; these changes may be, at least in part, due to a reduction in the number of TH+ fibers in Sp5C that we observed in these mice. The LC-evoked inhibition was due to the activation of the α2-NA receptors because it was reduced by the receptor antagonist yohimbine. The reduction in LC inhibition in the Sp5C neurons could explain the presence of pain in the orofacial area that these diabetic mice presented. Therefore, this reduction in inhibition could favor the development of chronic pain in diabetes.

In the present study, LC stimulation induced an inhibition of vibrissal responses that was mainly due to the activation of the α2-NA receptors because it had been blocked by the antagonist yohimbine, as also seen in the spinal cord (Jones, 1991; West et al., 1993; Sonohata et al., 2004). The activation of α2-NA receptors directly reduces pain transmission by reducing transmitter release of substance P and glutamate from the primary afferent terminals and by hyperpolarizing the membrane potential of spinal neurons *via* G-protein mediated activation of potassium channels (Kamisaki et al., 1992, 1993; Sonohata et al., 2004). However, in the present results, LC-evoked inhibition was not increased by the non-selective α2-NA agonist clonidine in the control mice. This unexpected result may be due to the fact that most of the α2-NA receptors were activated by the LC stimulation. By contrast, LC-evoked inhibition was reduced in the diabetic mice, and in this case, clonidine was able to increase the inhibition (see below).

It is interesting to note that LC stimulation induced facilitation of the tactile responses in the somatosensory thalamus and cortex (Waterhouse et al., 1998; Devilbiss and Waterhouse, 2004; Moxon et al., 2007). By contrast, LC inhibits somatosensory responses in the spinal dorsal horn (Jones, 1991; West et al., 1993; Sonohata et al., 2004) and in the trigeminal nucleus (Sasa and Takaori, 1973; Tsuruoka et al., 2003) and present results. The distinct effect of the LC projections may be due to the type of NA receptors that are activated. While α2-NA receptors mediate the inhibition of nociception in many acute pain models through descending pathways, the α1-NA receptor signaling in the medial prefrontal cortex contributes to chronic pain facilitation (Kaushal et al., 2016).

Neuropathic pain is one of the most prevalent complications of diabetes (Vincent et al., 2011; Feldman et al., 2019). In the present study, the diabetic mice showed a reduced threshold for evoking escape behavior by the mechanical stimulation of the vibrissal pad indicating that they suffered chronic pain in the trigeminal territory. It is known that the number of intraepidermal nerve fibers in diabetic subjects is decreased compared with age-matched nondiabetic healthy control subjects, and diabetic subjects have shorter nerves, which often end bluntly at the dermal surface of the basement membrane (Shun et al., 2004). Therefore, these data suggest that chronic pain in diabetic patients may be due to peripheral nerve degeneration. Our study cannot rule out this possibility; however, our data showed that the descending inhibitory pathway from LC was reduced in diabetic patients. This reduction may be due to a decrease in the TH+ LC neurons as well as in the TH+ stained fibers in the Sp5C of diabetic mice, as is shown in Figure 1. A tendency toward the reduction of the TG neurons has been also observed (Figure 1F). It is known that TH+ neurons exist in the ganglion although their function is not well established (Lallemend and Ernfors, 2012; Usoskin et al., 2015; Brumovsky, 2016). This reduction of TH+ ganglion neurons may be due to the peripheral nerve degeneration described above. However, the fact that in our experiments, both terminals of lamina II, where inputs from the periphery end, and those of laminae III–IV, whose fibers come from other nuclei, were reduced, suggesting that most of the TH+ fibers reduced in diabetic animals come from the LC. A further study about the involvement of the TG in these processes should be necessary.

The LC nucleus plays an important role in descending pain control (Jones, 1991; Millan, 2002; Hayashida et al., 2008; Hickey et al., 2014). Accordingly, the LC-evoked inhibition of vibrissal responses is reduced in diabetic mice, indicating that the descending NA inhibitory system from LC may be reduced in them. Although our findings suggest a reduction in the NA inputs to Sp5C in the diabetic mice, their α2-NA receptors remained active in Sp5C neurons since the LC-evoked inhibition could be increased in the diabetic mice when the α2-NA receptor agonist clonidine was injected intraperitoneally. These data suggest drugs that activate α2-NA receptors, such as clonidine, may have a therapeutic effect in relieving chronic pain.

Several results have suggested that LC-evoked inhibition of Sp5C neurons was not only elicited by NA inputs: (1) yohimbine reduced but did not block LC-evoked orthodromic responses in the Sp5C neurons and (2) yohimbine reduced LC-evoked inhibition but the vibrissal responses were also reduced rather than an expected increase in their responses due to yohimbine-induced disinhibition. These findings suggest that LC stimulation may also inhibit Sp5C activity by the activation of inhibitory interneurons. GABAergic and glycinergic neurons modulate the synaptic transmission in the Sp5C and their activity changes after the induction of pain by infraorbital chronic constriction injury (Garcia-Magro et al., 2020). Our findings suggest that the balance between the direct inhibition evoked by the LC through α2-NA receptors and an indirect inhibition evoked by the activation of GABAergic and/or glycinergic neurons could be altered in diabetic animals. Further experiments must be performed to determine how the LC innervates inhibitory Sp5C cells.

Recently, it has been reported that the induction of neuropathic pain through chronic constriction injury induces morphological changes in the LC (Bravo et al., 2022; Camarena-Delgado et al., 2022). An increase in NA immunoreactivity in the spinal dorsal horn was reported after nerve injury (Ma and Eisenach, 2003), suggesting that a gradual enhancement in the NA inputs may act as an adaptive mechanism to counteract chronic pain. However, our findings in the diabetic mice showed a reduction in the LC-evoked inhibition and a reduction in the NA inputs to Sp5C that could facilitate the development of chronic pain, as was stated by our behavioral studies. Models of chronic pain elicited by nerve injury or diabetes are not similar. Infraorbital chronic constriction injury in rats induces an increase in the excitability of Sp5C neurons as was observed by an increase in spontaneous activity and responses to light tactile stimulation of the vibrissal (Martin et al., 2010; Garcia-Magro et al., 2020). Equally, an increase in excitability occurs in the lumbar spinal cord after sciatic constriction or spinal nerve ligation (Mansikka and Pertovaara, 1997; Balasubramanyan et al., 2006; Pertovaara, 2006). By contrast, the diabetic mice did not display an increase in their excitability because the spontaneous activity (data not shown because the Sp5C neurons were silent in both the control or diabetic mice) or the vibrissal responses (2.4 ± 0.08 spikes/stimulus in the control mice vs. 2.5 ± 0.07 spikes/stimulus in the diabetic mice) did not change. These contradictory results may be due to the mechanism used to induce pain. In our mouse model, a lesion of the pancreas by STZ induced a decrease in some growth factors, such as insulin and IGF-I that may reduce LC neuronal activity. Specifically, our research group has reported that a reduction in IGF-I levels in aging or APP/PS1 animal models of Alzheimer's disease induced an improvement in the neuronal activity in different brain areas (Zegarra-Valdivia et al., 2019, 2021; Garcia-Magro et al., 2022). Our results suggest that a reduction in IGF-I could reduce the descending inhibitory systems, facilitating the development of pain.

In conclusion, our findings suggest that a reduction in NA inputs in the Sp5C of diabetic mice may contribute to the development of trigeminal chronic pain by reducing the LC-evoked inhibition produced by α2-NA receptor activation. A deeper knowledge of these mechanisms will help to identify new therapeutic targets and improve treatments for diabetic subjects.

## Data availability statement

The raw data supporting the conclusions of this article will be made available by the authors, without undue reservation.

## Ethics statement

The animal study was reviewed and approved by Autonomous University of Madrid and Government of the Community of Madrid; PROEX: 181.6/21.

## Author contributions

AM-L and NG-M conducted experiments, analyzed electrophysiological and behavioral experiments, and conducted immunohistochemistry studies. YM and AN designed and conducted experiments. All authors have read and agreed to the published version of the manuscript.
